# LDkit: a parallel computing toolkit for linkage disequilibrium analysis

**DOI:** 10.1186/s12859-020-03754-5

**Published:** 2020-10-16

**Authors:** You Tang, Zhuo Li, Chao Wang, Yuxin Liu, Helong Yu, Aoxue Wang, Yao Zhou

**Affiliations:** 1 Electrical and Information Engineering College, Jilin Agricultural Science and Technology University, Jilin, China; 2 Shenzhen Branch, Guangdong Laboratory for Lingnan Modern Agriculture; Genome Analysis Laboratory of the Ministry of Agriculture; Agricultural Genomics Institute at Shenzhen, Chinese Academy of Agricultural Sciences, Shenzhen, China; 3 College of Horticulture and Landscape Architecture, Northeast Agricultural University, Harbin, China; 4 Key Laboratory of Crop Biotechnology Breeding of the Ministry of Agriculture, Beidahuang Kenfeng Seed Co., Ltd., Harbin, China; 5 Information Technology Academy, Jilin Agricultural University, Changchun, China

**Keywords:** Population genetics, Parallel computing, Graphical user interface, Linkage disequilibrium

## Abstract

**Background:**

Linkage disequilibrium (LD) analysis is broadly utilized in genetics to understand the evolutionary and demographic history and helps geneticists identify genes associated with interested inherited traits, such as diseases. There are some tools for linkage disequilibrium analysis either in a local or online way; however, there has been no such tool supporting both graphical user interface (GUI) and parallel computing.

**Results:**

We developed a GUI software called LDkit for LD analysis, which supports parallel computing. The LDkit supports both variant call format (VCF) and PLINK ‘ped + map’ format. At the same time, users could also just analyze a subset of individuals from the whole population. The LDkit reads the data by block and then paralleled the computation process by monitoring the usage of processes. Assessment on the Human 1000 genome data showed that when paralleled with 32 threads, the running time was reduced to less than 6 minutes from ~77 minutes using the chromosome 22 dataset with 1,103,547 SNPs and 2504 individuals.

**Conclusions:**

The software LDkit can be effectively used to calculate and plot LD decay, LD block, and linkage disequilibrium analysis between a site and a given region. Most importantly, both graphical user interface (GUI) and stand-alone packages are available for users’ convenience. LDkit was written in JAVA language under cross-platform support.

## Background

Most of the population genetic analyses, such as detection of sites under selection, estimation of divergence time between different populations, positional cloning, and genome wide association studies (GWAS) are based on linkage disequilibrium (LD) theory, which has been investigated over 100 Years [1]. For example, the degree and pattern of LD in different human populations were investigated to facilitate positional cloning [2]; currently, this analysis has been replaced by GWAS when large markers are available [3, 4]. The decay of LD reflected the historical recombination of population, and LD blocks could be used to represent the patterns of LD and recombination hotspots [5].

As there is growing interest in LD analysis, some software tools are available to provide analysis of LD decay and/or LD block, such as the LDlink, an online application designed for human genetics [6, 7]. The latest version of PLINK could be used for calculating LD through extensive use of bit-level parallelism, supporting both multicore and cluster parallelism [8, 9]. PLINK provides the most statistic for LD analysis, such as r, r^2^, D prime, and signed D prime; however, PLINK is not designed for LD analysis on purpose and has many inconveniences, for example, the output of PLINK is the pair-wise linkage result and takes up considerable storage space. Users need to analyze the large file further to visualize the pattern of LD decay. There is other software designed for LD analysis, like the recently published software PopLDdecay, which was designed to support the variant call format (VCF) and subgroup analysis; however, it does not support parallelism and could not be used for LD block analysis [10]. LD analysis is time-consuming when there are a large number of variants and sample size. Although users could perform LD analysis on each chromosome to manually achieve parallelism, the analysis for large genome species with long chromosome length is still challenging. For example, the length of the wheat 3B chromosome is about 830 million base pairs, which is more than twice the size of the rice genome [11, 12]. Therefore, LD analysis of wheat populations using PopLDdecay may still take about one day to finish.

Here, we developed a package LDkit to provide an effective tool for LD analysis. For the convenience of users, we offer both a graphical user interface (GUI) that can be used on a personal computer and a stand-alone package on a cluster or cloud platform. The most common analysis, such as LD decay and LD block, is supported in the LDkit. In some situations, we are also interested in verifying whether a single variant is in linkage disequilibrium to the other or not, but no tool is available currently, which is accommodated as LD site in the LDkit.

## Implementation

**Fig. 1:**
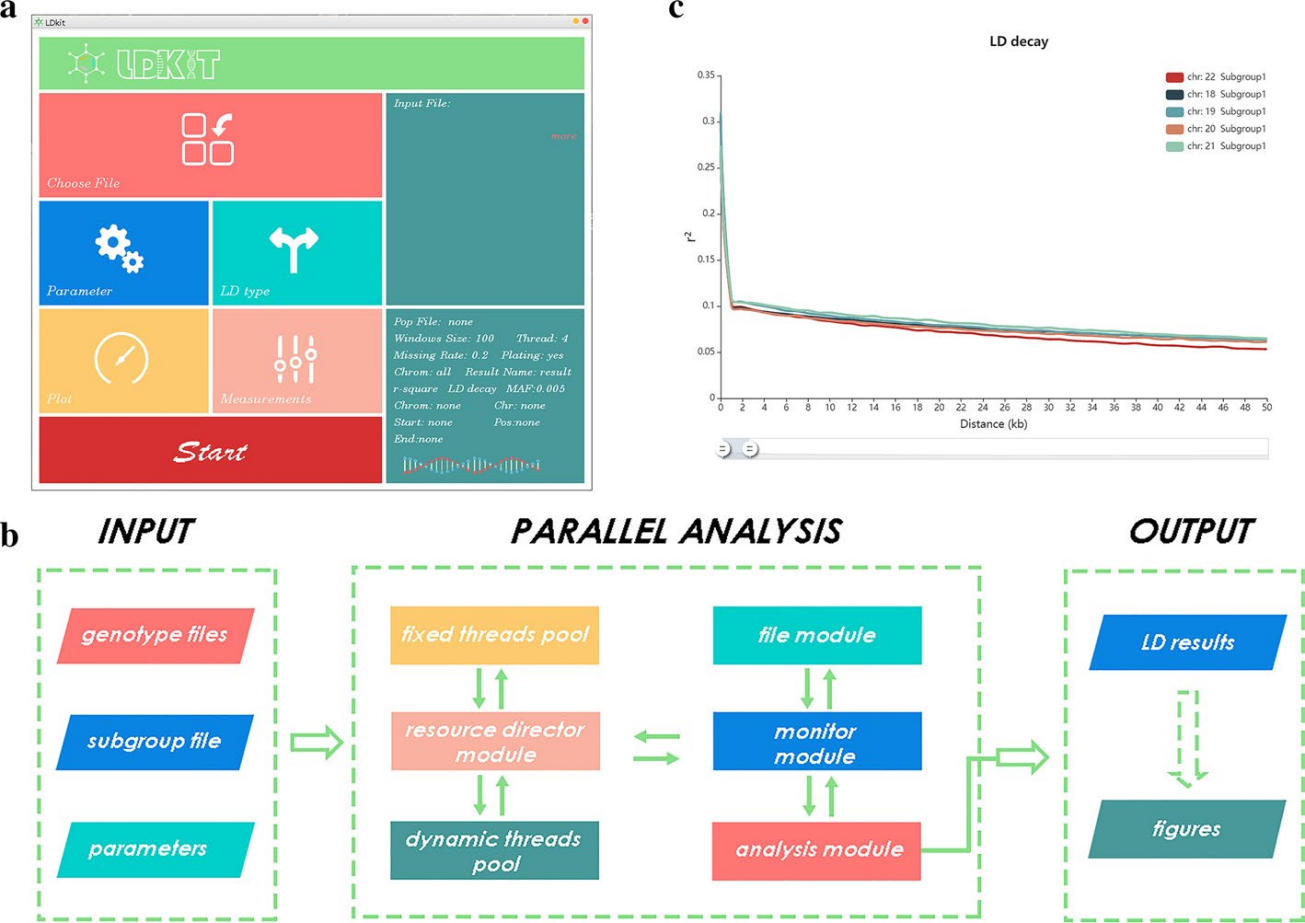
The LD decay analysis in LDkit. **a** The layout of the interface was designed according to the process of LD analysis. **b** The workflow of parallel analysis. **c** The figure of LD decay plotted by LDkit

LDkit is developed in JAVA with supporting of parallel computing (Fig. 1a). We also designed the software with the advantages of other software such as PopLDdecay [10] and Haploview [13]. For example, LDkit supports both VCF and PLINK formats and could calculate the LD decay in a subpopulation as PopLDdecay. However, PopLDdecay does not support the LD block analysis, which is the main feature in Haploview. We designed LDkit supporting both LD decay and LD block analysis as Haploview. We also implemented the LD site analysis, which makes LDkit different from other tools. The results could be visualized directly in LDkit or through other software.

### Data preparation

LDkit accepts two widely used formats. The input file could be either VCF format or PLINK ‘ped + map’ format [8]. LDkit is programmed using parallel computing technology so that there is no need to separate files by chromosomes. For minimizing the analysis steps when there are multiple subgroups, all subgroup information could be given in a single file, and the LD analysis for all subgroups will be performed at one-time submission.

### LD analysis

Two types of LD measurements D’ and r^2^ were implemented in LDkit [14, 15]. The statistic D’ is calculated using the same algorithm in PopLDdecay. Instead of the r^2^ derived from D’ statistic as implemented in Haploview and PopLDdecay, the LDkit takes the squared Pearson correlation as PLINK (with –r2 flag). There are three different kinds of LD analyses supported by LDkit. In addition to supporting the most used LD decay and LD block analysis, LDkit also implemented a function to calculate linkage between a site and a given region, which is called the LD site.

### Parallel computing

As the multicore machine is easily available currently, we parallelized the computation in a multiple-threads way with specific optimization on programing (Fig. 1b). Firstly, the whole data will be read into memory and filtered at the same time. This step is currently not parallelized. Then, the entire data will be divided into N*2 parts, *N* refers to the number of all available threads in the machine. Alternatively, the number of threads could be given by users to avoid the potential overuse of the computer resource. To reduce the channel blackout periods in this step, we optimized the program by balancing each thread’s usage using multiple virtual threads. Finally, the partial results from all partitions are gathered and combined into the final output.

### Visualization and output

LDkit could output the figure of LD decay or heatmap of the LD block directly. Different from other software, LDkit provides an adjustable view of LD decay to help users choose a better combination of parameters (Fig. 1c). Users might want to plot the LD decay; accordingly, the initial results for plotting are provided by LDkit. It will be convenient for users to represent the result with other software such as R or python.

## Results and discussion

### LDkit interface

The main interface of LDkit is designed to include all sections, such as file inputs, parameter setting, selection of LD type and measurement, and plotting. All settings will be displayed on the right side for the user to double-check before running. Considering the long running time of large files, we designed a DNA logo in the bottom-right interface. The dynamic DNA logo indicates the working status of the task. Considering the multiple-task situation, we also developed a stand-alone package that could be used locally or on a cloud platform.

### LD decay analysis

To assess the performance of LDkit on LD decay analysis, we tested the software using the chromosome 22 dataset from the Human 1000 genome. There are 2,504 individuals and 219,790 variants in this dataset. For LD decay analysis, we only retained single nucleotide polymorphisms (SNPs) with a missing rate below 0.2 and an allele frequency higher than 0.01. The window size used to calculate pair-wise r^2^ was set to 100 kb. Although the algorithms for calculating the r^2^ in LDkit and PopLDdecay are different, the two software generated the same pattern of LD decay (Additional file 1: Fig. S1). When only one thread was used, PopLDdecay is about 1 - 4 times faster than LDkit with almost the same memory usage. LDkit spent about 77 minutes, nearly twice as long as PopLDdecay (Table 1). When the number of threads was set to 32, the runtime was reduced to less than 6 minutes. The memory used was not affected by the increasing number of threads and was comparable with PopLDdecay.

**Table 1:** Comparison of performance on LD decay using PopLDdecay and LDkit

Population	Chromosome	Number of individuals	Number of SNPs	Peak memory (GB)		Running time (Minute)	
				PopLDdecay	LDkit	PopLDdecay	LDkit
CEU	Chr22	183	1,103,547	9.87	10.33	2.15	10.36
CHS	Chr22	171	1,103,547	9.1	10.12	2.03	11.11
ESN	Chr22	173	1,103,547	9.36	10.25	2.09	10.88
GWD	Chr22	180	1,103,547	9.74	10.45	2.11	10.01
IBS	Chr22	162	1,103,547	9.2	10.03	2.01	10.93
All	Chr22	2504	1,103,547	10.59	11	46.31	77.47

To systematically evaluate the performance of LDkit, we tested the running time of LDkit using different subpopulations under a various number of threads (Additional file 2: Table S1). We noticed that the running times on populations with similar population size were almost the same when using the same threads and the running time was reduced with the increasing number of threads, but the running time was not exactly linear with the number of threads or population sizes due to the time on resource monitoring. For example, for the IBS population with 162 individuals, the running time is about 11 minutes with one thread and decreased to about half a minute with 32 threads (Table 2). We also observed that the running time is longer using 64 threads than that using 32 threads in a small population, while the running time decreases as the number of threads increases in a large population (Additional file 2: Table S1). We also compared the performance with PLINK under multiple threads situation (Table 2). Using the same IBS population, PLINK took only one-fifth of the memory usage and 1.3–7.5 times faster than LDkit using different threads. However, only the time for calculating the pair-wise LD analysis was considered as PLINK does not support the following analysis.

**Table 2:** Comparison of multiple-threads performance using PLINK and LDkit

Number of threads	Peak memory (GB)		Running time (Minute)		File sizes (GB)	
	PLINK 2.0	LDkit	PLINK 2.0	LDkit	PLINK 2.0	LDkit
1	1.05	10.03	0.44	10.93	13.97	0.0019
4	1.05	10.63	0.43	3.14	13.97	0.0019
8	1.05	10.66	0.42	1.95	13.97	0.0019
16	1.05	11.01	0.42	1.08	13.97	0.0019
32	1.05	11.13	0.41	0.53	13.97	0.0019
64	1.05	11.25	0.43	0.63	13.97	0.0019

**Table 3:** Comparison of LDkit and Haploview using MHC region

Population	Number of individuals	Number of SNPs	Peak memory (GB)		Running time (Minute)	
			LDkit	Haploview	LDkit	Haploview
All	2504	97,900	10.13	1.00	9.20	200.00
CEU	183	97,900	10.66	1.00	4.90	110.00
CHS	171	97,900	10.99	1.00	4.70	105.00
ESN	173	97,900	11.22	1.00	5.10	101.00
GWD	180	97,900	11.19	1.00	4.90	103.00
IBS	162	97,900	11.28	1.00	4.80	100.00

### LD block analysis

**Fig. 2:**
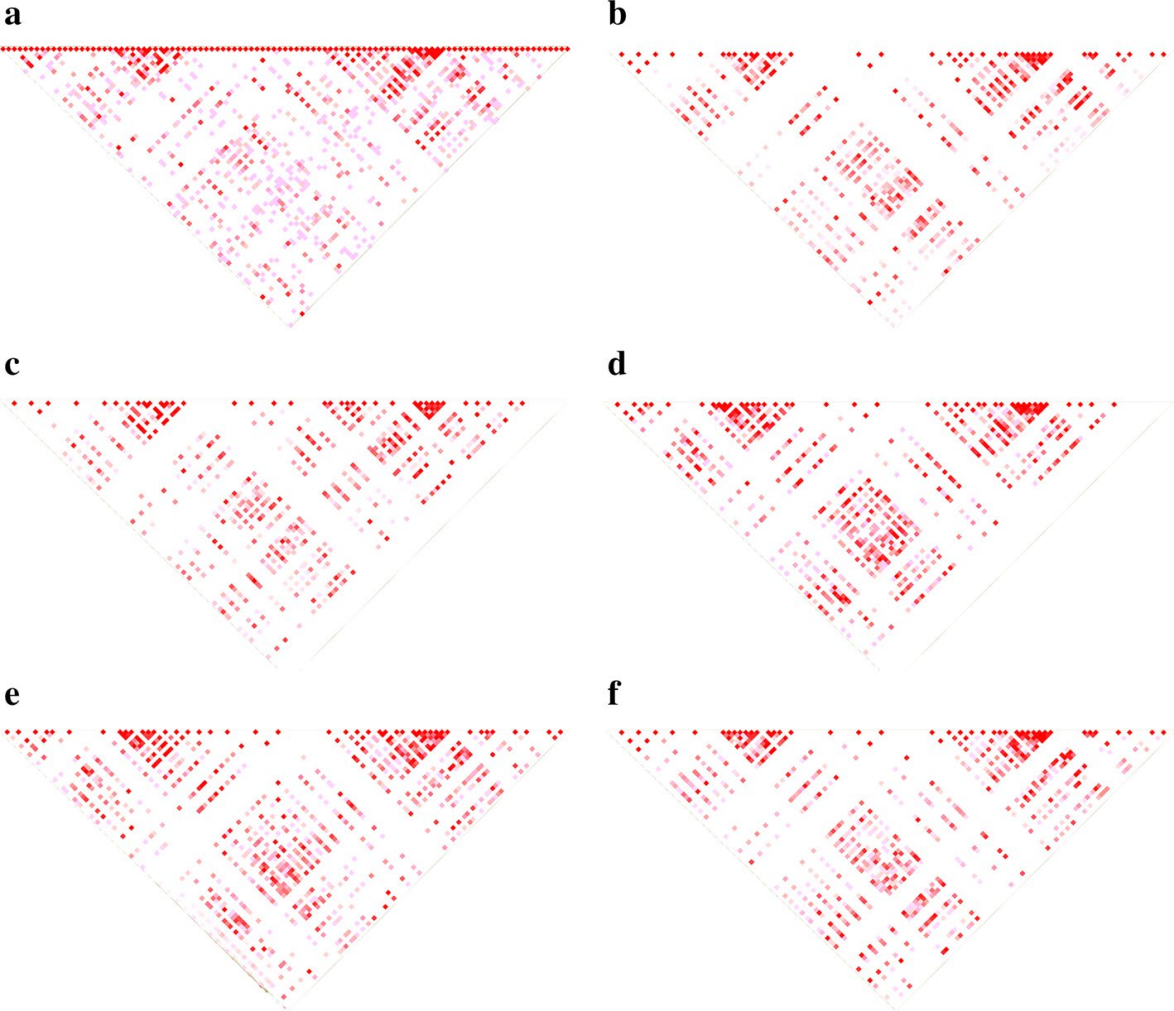
LD block analysis of the MHC region in different groups using LDkit. **a**–**f** All, CEU, CHS, ESN, GWD, and IBS

Both Haploview and LDkit provide the GUI and stand-alone package at the same time; the comparison of LD block could be performed under the personal computer using the GUI package. We used the MHC region (Chr6: 28,954,920-32,041,234) in the human genome for the evaluation of LD block analysis (Fig. 2). Compared with Haploview, which is also a java program, LDkit performances much better at running time with more memory usage. For example, in the case of single-thread using, the running time of LDkit is less than 5 minutes, about 19 times faster than Haploview, while the memory usage has increased from 1 Gb to 10 Gb for IBS population with 162 individuals (Table 3). We also evaluated the performance of LD block analysis using different population sizes. When tested using the whole population with 2,504 individuals, the running time of Haploview was about 200 minutes, while it was only 9.2 minutes for LDkit. For memory usage, the Haploview only needed about 1Gb memory, while the memory required by LDkit ranged from 10.13 to 11.28 Gb.

### LD site analysis

Both LD decay and LD block only consider the linkage for local sites, while the LD site could be considered as the global linkage analysis. For example, the linkage between two paralogs located in different chromosomes. Currently, we only realized the function for calculating the linkage between one site and one region, which is called the LD site in LDkit. Considering the real situation of this function, LDkit generates linkage results in text format and could be plotted in other software like R and python.

## Conclusions

LDkit is a user-friendly JAVA software that could be used for LD analysis across multiple platforms. It was designed to provide an easy-to-use and fast tool for species with a large genome. We achieved the LDkit with both GUI and stand-alone packages supporting parallel computing. LDkit has conducted parallel computing programming to improve analysis efficiency and is comparable with other tools evaluated using the Human 1000 genome dataset. There are three functions (LD decay, LD block, and LD site) and two measurements (r^2^ and D’) implemented in the LDkit, making it valuable under most of the LD analysis scenarios. Although LDkit has significant improvement in many parts, it performs better than the Haploview, which is also programmed using the JAVA language, other tools programmed with C/C++ performed much better than LDkit. The running time of LDkit is longer than PopLDdecay and PLINK at the same threads. However, LDkit provides the GUI package, making it easier to use compared with PopLDdecay and PLINK on a personal computer.

Furthermore, LDkit is much more efficient than PopLDdecay under a multiple-threads model. Although LDkit requires nine times more memory than Haploview for LD block analysis for the testing data, it is about 19 times faster at running time, and most importantly, the memory needed by LDkit could be satisfied in multicore machines under most situations, promising its application in the future. We offer the LDkit as an alternative tool for users who desire to perform the LD analysis in a fast way when a multicore machine is available.

## Availability and requirements

- Project name: LDkit
- Project home page: https://github.com/tangyou79/LDkit
- Operating system(s): Linux, Mac or Windows
- Programming language: java
- Other requirements: Java 1.8 or above
- License: GNU GPL
- Any restrictions to use by non-academics: LDkit is free and open to all users

## Supplementary information

**Additional file 1: Fig. S1** Comparison of LD decay results between LDkit and PopLDdecay.

**Additional file 2: Table S1** Running time using different parameters. **Table S2** Comparison of LD statistics in different software. **Table S3** Simulated genotypes for comparison of different software.

## Data Availability

LDkit is open-source software. Both GUI and stand-alone packages of LDkit are freely available at GitHub (https://github.com/tangyou79/LDkit).

## Abbreviations

LD: Linkage disequilibrium
GUI: Graphical user interface
GWAS: Genome wide association studies
CEU: Utah Residents (CEPH) with Northern and Western European Ancestry
CHS: Southern Han Chinese
ESN: Esan in Nigeria
GWD: Gambian in Western Divisions in the Gambia
IBS: Iberian Population in Spain
EAS: East Asian
MHC: Major histocompatibility complex
