# Supplementary figures and images for: LDkit: a parallel computing toolkit for linkage disequilibrium analysis
Source: BMC Bioinformatics. 2020 Oct 16;21:461. doi: 10.1186/s12859-020-03754-5 (PMC7565767; doi:10.1186/s12859-020-03754-5)

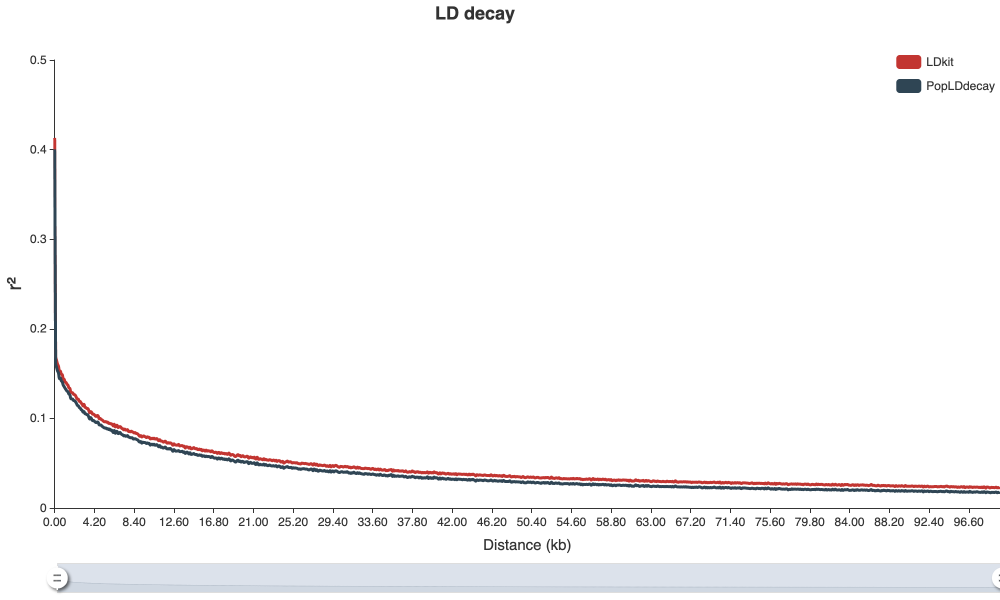


**Figure S1.** **The comparison of LD decay results between LDkit and PopLDdecay.**

Supplement: Supplementary file 1 — Additional file 1: Fig. S1 Comparison of LD decay results between LDkit and PopLDdecay. [file 12859_2020_3754_MOESM1_ESM.docx]
